# Effects of Severe Plastic Deformation and Ultrasonic Treatment on the Structure, Strength, and Corrosion Resistance of Mg-Al-Zn Alloy

**DOI:** 10.3390/ma15207200

**Published:** 2022-10-15

**Authors:** Denis A. Aksenov, Ayrat A. Nazarov, Georgiy I. Raab, Arseniy G. Raab, Elvira I. Fakhretdinova, Rashid N. Asfandiyarov, Maria A. Shishkunova, Yulia R. Sementeeva

**Affiliations:** 1 Institute of Molecule and Crystal Physics UFRC RAS, 151 pr. Oktyabrya, Ufa 450075, Russia; 2 Institute of Physics of Advanced Materials, Ufa State Aviation Technical University, 12 Karl Marx, Ufa 450001, Russia; 3 Institute for Metals Superplasticity Problems RAS, 39 S. Khalturin St., Ufa 450001, Russia; 4 Laboratory “Mechanics of Gradient Nanomaterials” A.P. Zhilyaeva, Nosov Magnitogorsk State Technical University, 38 pr. Lenina, Magnitogorsk 455000, Russia

**Keywords:** magnesium alloys, ECAP, ultrasound, corrosion resistance, strength, special boundaries

## Abstract

Nowadays, there is a great demand for increasing the strength and corrosion resistance of magnesium alloys for their wider use in machine engineering, oil industry, and medicine. This paper is devoted to a study on the effects of the combined process of reduction and equal channel angular pressing, as well as the subsequent ultrasonic irradiation on the structure, strength, and corrosion properties of the Mg-Al-Zn alloy. Deformation processing results in an increase of the strength up to 280 ± 10 MPa. A fine-grained structure is formed with a grain size of 10–20 µm and small recrystallized grains 1–2 µm in size. The corrosion resistance in the HCl medium falls down significantly. Action of ultrasound on the deformed specimen leads to an increased fraction of high-angle boundaries, in particular, the fractions of special, fully overlapping Σ13a boundaries and twin boundaries of Σ15b and Σ17a systems. Due to the ultrasonic treatment, the strength of the Mg-Al-Zn alloy increases up to 310 ± 5 MPa, while the corrosion resistance in HCl almost doubles.

## 1. Introduction

Due to their light weight, high specific strength, rigidity, and machinability, magnesium and its alloys are widely used in various industries. Magnesium alloys are non-magnetic and have a sufficient heat conductivity and vibration stability. Most extracted magnesium is used for the production of magnesium structural alloys. However, much attention is paid today to the use of magnesium alloys in medicine, namely as a material for manufacturing bio-resorbable implants [1,2]. Another use of magnesium alloys is in the oil industry; for example, the Mg-Al-Zn alloy is used to manufacture temporary seals [3].

Despite all the advantages, magnesium and its alloys are characterized by low strength and corrosion resistance, and these disadvantages substantially limit their application. Today, much attention is paid to strengthen magnesium and its alloys using the methods based on severe plastic deformation (SPD) [4]. Magnesium and its alloys are poorly deformable because they have a hexagonal close-packed lattice and only two primary slip systems: (0001)<$11\bar{2}0$> and ($10\bar{1}0$)<$11\bar{2}0$> [5]. To increase the number of possible slip systems, it is usually required to increase the deformation processing temperature, which has a natural effect on the level of resulting properties. The latest trend is to decrease the deformation processing temperature to achieve the highest final strength [6,7,8].

Another negative effect of deformation processing in general and SPD in particular is an increase in the degradation rate of magnesium materials in corrosion environments. As noted in [9], the work dedicated to the SPD of magnesium alloys, corrosion in this case, occurs as pitting corrosion mainly developing on crystal lattice defects. Grain boundaries greatly contribute to the development of corrosion. Being areas of high structural non-equilibrium, they can substantially accelerate the process of metal corrosion, especially after deformation processing [10,11]. Particles of the second phases can also promote corrosion development [12]. It is known that SPD methods can promote dynamic aging when the volume fraction of second-phase precipitates increases [13,14,15].

Heat treatment below the recrystallization point is used to stabilize a non-equilibrium structure obtained by deformation methods. This approach helps to increase the ductility of materials, but also causes grain growth, which has a negative effect on the strength characteristics of the material [16].

Ultrasonic irradiation, or ultrasonic treatment (UST), is an alternative method which allows one to reduce the level of structural non-equilibrium in deformed metals [17]. In general, the action of ultrasonic waves can result in various structural changes in materials depending on the parameters of ultrasound and initial state of material. These changes are caused by oscillating elastic stresses induced by ultrasound, which exert forces acting on crystal lattice defects such as vacancies, atoms of alloying and trace elements, dislocations, and grain boundaries. Early experiments showed that high-intensity ultrasonic vibrations resulted in an increase in the concentration of vacancies and rapid multiplication of dislocations in crystals [18,19,20]. More recently, experimental studies on the effect of UST on the structure and mechanical properties of ultrafine grained (UFG) Ni processed by SPD were carried out [21,22,23]. These studies have shown that the UST results in a stabilization of the non-equilibrium structure of SPD-processed Ni, relief of internal stresses, enhancement of the thermal stability of microstructure, and a simultaneous increase in the strength and ductility of ECAP-processed UFG Ni. Simulations carried out in [24,25] using the discrete-dislocation approach showed that ultrasonic treatment had a significant effect on the dislocation structure. It was noted that at low amplitudes of the ultrasonic wave, dislocation walls were built inside the grains, while at large amplitudes, the dislocation substructure was destroyed and most of the lattice dislocations were absorbed by the grain boundaries.

Recently, significant interest has been given to the use of the acoustoplastic effect, which occurs during ultrasonic processing [26,27,28,29,30]. For instance, the application of ultrasonic action to the tool during the deformation process significantly reduces the stress level required to deform a sample, which makes it possible to use a softer technological processing mode, for example, with a decrease in the deformation processing temperature. It is also worth noting that the method of surface ultrasonic finishing [31,32] can significantly improve the mechanical and operational characteristics of the surface to a depth of up to 75 microns [31].

This paper is aimed to find out specific features of the effects of post-deformation UST on the grain and dislocation structure of the Mg-Al-Zn alloy subjected to SPD by combining the methods of reduction and equal channel angular pressing (ECAP) that can be used in order to improve strength characteristics and corrosion resistance.

## 2. Materials and Methods

**Figure 1:**
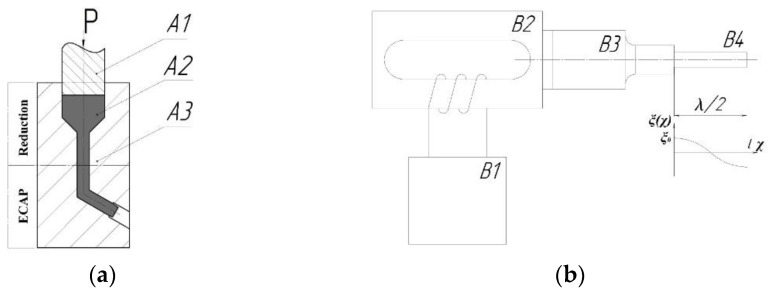
Layout of deformation processing (**a**): A1—plunger; A2—specimen; A3—die. Layout of ultrasound post-deformation processing (**b**): B1—ultrasonic generator, B2—ultrasonic transducer, B3—waveguide, B4—half-wave length sample.

An experimental magnesium alloy of Mg-Al-Zn was selected as a material for studies (Mg-5.1 Al-2.5 Zn wt.%). The closest equivalent of this alloy used in production is the deformable AZ61 alloy. The initial commercially supplied material (processed by rolling) represented cylindric workpieces having the diameter of 40 mm and length of 80 mm. The initial workpieces were deformed by combining the reduction and ECAP. A special matrix with two deformation sites was manufactured, one for reduction to a diameter of 16 mm, and the other for ECAP with an angle of 120° between the channels (Figure 1a). Deformation was carried out at 350 °C up to the total accumulated strain e ≈ 2. The rate of deformation was equal to 10 mm/s. The first cycle of such combined deformation processing resulted in rod-shaped specimens 16 mm in diameter. These specimens were then subjected to bulk UST by applying the method used in [22,23]. The essence of this method consists of the excitation of a standing ultrasonic wave in a sample. For this, the specimen should represent a bar, either cylinder or parallelepiped shaped, with a length, *l*, equal to the half-wavelength of ultrasound, λ/2, for the given value of frequency, *f*. This bar is tightly screwed to the waveguide of a vibrating system schematically illustrated in Figure 1b. Ultrasonic transducer B2 powered by ultrasonic generator B1 excites longitudinal vibrations which are transferred to sample B4 by waveguide B3. At the resonance frequency *f*, a standing longitudinal wave is created in the whole vibrating system. This wave induces oscillating normal strains and stresses in the specimen.

The distribution of the amplitudes of displacements, ξ_0_, along the specimen obeys the law [22]

(1) $$\xi =\xi_{0}\cos\frac{2\pi }{\lambda }x,$$

where *x* is the distance from the screwed end of the sample. This distribution is represented by a schematic graph below the sample in Figure 1b.

The amplitudes of the normal elastic strains and stresses induced in the displacement field given by Equation (1) are calculated, respectively, as ε(x)=|dξ(d)/dx| and σ(x)=Eε(x), where *E* is the Young modulus of the metal, i.e.,

(2) $$\varepsilon \left(x\right)=\varepsilon_{0}\sin\frac{2\pi }{\lambda }x,\;\varepsilon_{0}=\frac{2\pi }{\lambda }\xi_{0}=\frac{\pi }{l}\xi_{0}$$

(3) $$\sigma \left(x\right)=\sigma_{0}\;sin\frac{2\pi }{\lambda }x,\;\sigma_{0}=\frac{2\pi }{\lambda }E\xi_{0}=\frac{\pi E\xi_{0}}{l}.$$

In the case of the magnesium alloy under study, the half-wavelength was experimentally determined to be equal to *l* = 86 mm for the resonance frequency of the vibrating system used, *f* = 19.8 kHz. The amplitude of vibrations at the specimen end was measured during experiments using a contactless capacity vibrometer [33] and was equal to ξ_0_ = 10 µm. Hence, the maximum amplitude of strains at the middle of the specimen was ε_0_ = πξ_0_/*l* = 3.7 × 10^−4^. The duration of UST was equal to *t* = 120 s.

The studies of the structure and properties were done in the longitudinal section of the specimen.

Structural studies were done using a JSM6490 focused beam microscope. Distribution maps of chemical elements were obtained using an Inca X-sight Oxford Instruments add-on for electron microprobe analysis. Electron backscatter diffraction (EBSD) maps were obtained using a TESCAN MIRA 3 LMH scanning microscope. The scanning step was 0.5 µm. The specimen surface was prepared using a Gatan Model 691 ion polishing system.

X-ray patterns were obtained using a Rigaku Ultima IV diffractometer under the Bragg-Brentano scheme by means of Cu Kα radiation generated at 40 kV and a current of 40 mA. Vertically (2/3 deg.) and horizontally (10 mm) limiting slits and a Soller slit 5° were used on the primary beam, and a vertically limiting slit (2/3 deg.), horizontal slit (0.6 mm), and Soller slit 5° were used on the reflected beam. A graphite monochromator was placed in front of the detector. X-ray patterns were obtained within the range from 35° to 145° in the continuous scanning regime with a rate of 0.25°/s.

The calculation of the lattice parameter, the size distribution of coherent scattering regions, the density of edge and helical dislocations, and the effective radius of dislocations was done by analyzing X-ray patterns under the whole powder pattern modeling approach implemented in the PM2K software [34].

Microhardness data for map building were obtained using an automatic hardness tester with an EMCO–Test DuraScan 50 image analysis system. Mechanical tests were done at room temperature using an Instron 8862 pull test machine at a strain rate of 10^−1^ s^−1^. For tensile tests, proportional cylindrical specimens with a working part diameter of 3 mm and an initial gage length of 15 mm were used.

Corrosion tests were carried out as per GOST 9.908-85. Concentrated HCl was used as a corrosion environment. The environment had the room temperature. Specimens were kept in the corrosion environment within 2 min. The weight of samples was measured using AR2140 analytical weights.

## 3. Results

**Figure 2:**
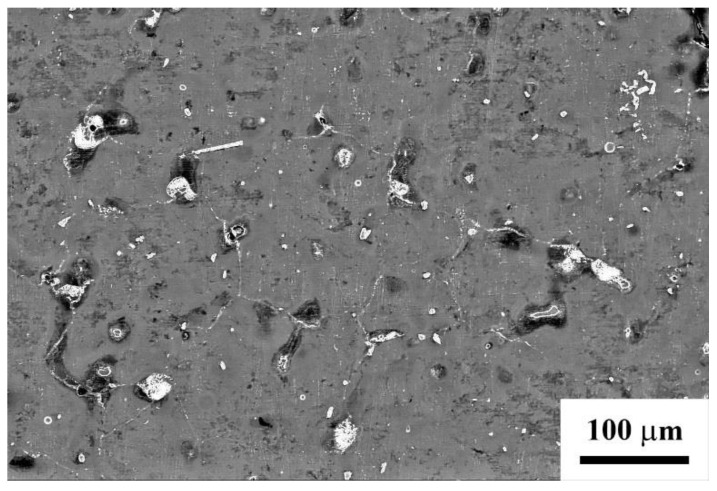
Structure of the initial state of Mg-Al-Zn alloy.

The material in the initial state had a coarse-grained structure with an average grain size of 110 ± 20 µm (Figure 2). The alloy had the microhardness of 440 ± 40 MPa and the tensile strength of 190 ± 15 MPa.

**Figure 3:**
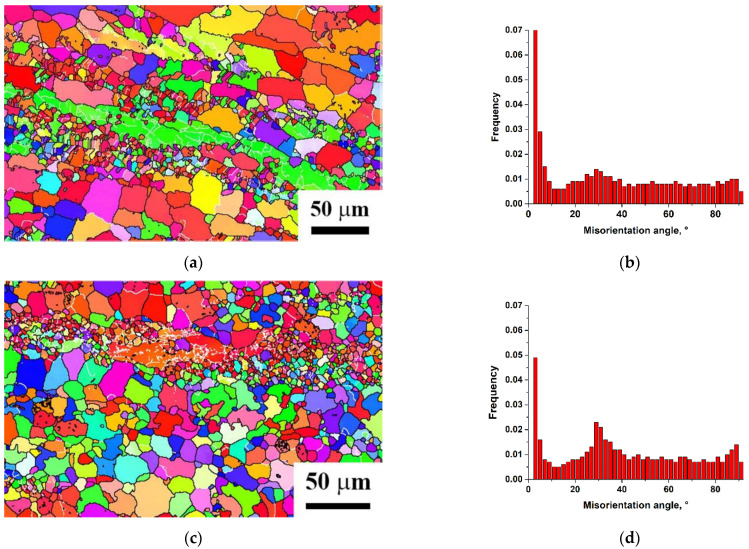
Maps and histograms of misorientations in the Mg-Al-Zn alloy after deformation processing (**a**,**b**) and post-deformation ultrasonic treatment (**c**,**d**).

The deformation of initial workpieces was done using a combined scheme including reduction and further ECAP (reduction–ECAP) at 350 °C and resulted in a structure of bimodal type: large deformed grains with sizes 10–20 µm were observed along with grains 1–2 µm in size, which were most likely formed during recrystallization (Figure 3a). This nature of recrystallization can be classified as the continuous one [35,36]. It should be noted that the combined deformation processing forms a structure with a high fraction of high angle boundaries, about 72%, while further UST leads to a further increase in the fraction of high angle boundaries up to 89% (Figure 3).

The X-ray structural analysis (Table 1) shows that an increase in the lattice parameter of the alloy and density of dislocations occurred as a result of deformation processing. The post-deformation UST resulted in a decrease in the lattice parameter and further increase in the density of dislocations. Changes in the lattice parameter after reduction with further ECAP and after post-deformation UST can be related to phase transformations stimulated by severe plastic deformation. Table 2 shows that deformation processing leads to a decreased volume fraction of second phases, which can be related to particle dissolution [13,37], while UST is followed by an insignificant increase in the total volume fraction of phases, which may be due to the decomposition of the solid solution. It should be noted that the latter is in agreement with the results of previous studies of processes of diffusion-controlled phase transitions in UST conditions [38].

**Figure 4:**
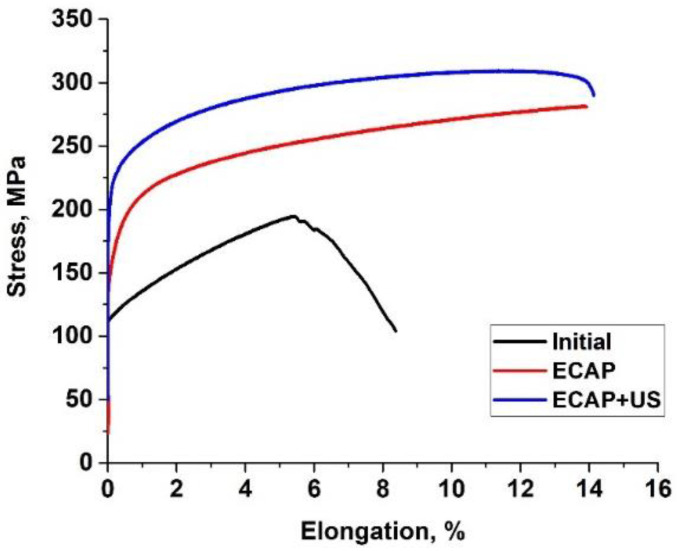
Hardening curves of Mg-Al-Zn alloy.

The structural refinement due to the processing by reduction–ECAP according to the proposed scheme resulted in a considerably enhanced strength of the alloy. The ultimate tensile strength increased from 190 ± 10 in the initial state up to 280 ± 10 MPa (Figure 4). UST led to an additional increase of the tensile strength which amounted now to 310 ± 5 MPa (Table 3). Moreover, the deformation processing under the proposed scheme led to an increase in the relative elongation to failure, which can be related to the formation of small recrystallized grains.

**Figure 5:**
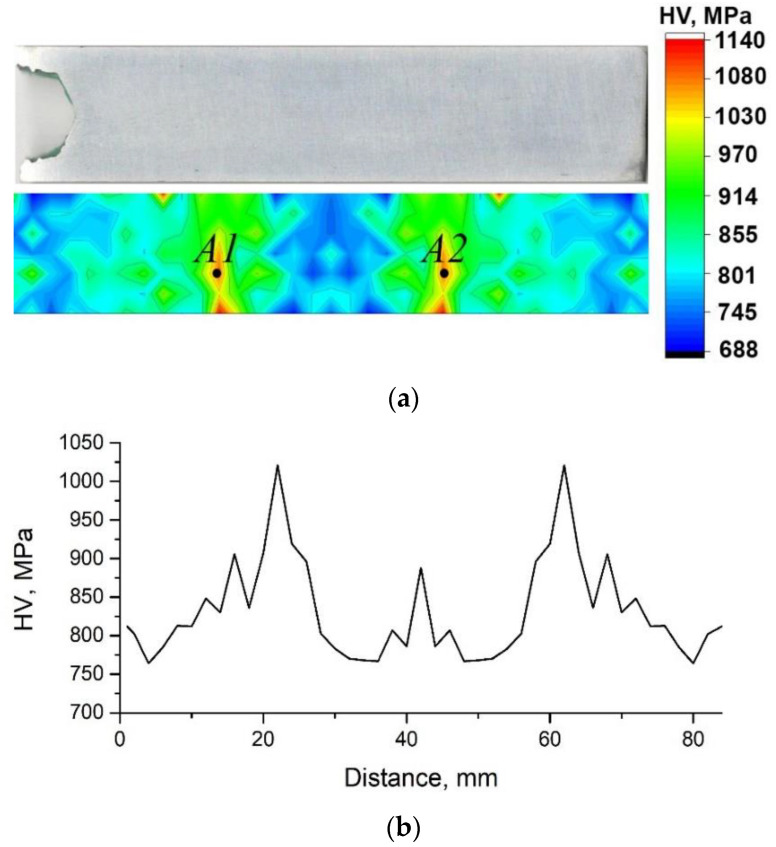
The photograph of the longitudinal section of the specimen and corresponding microhardness map of the Mg-Al-Zn alloy after reduction–ECAP and UST (**a**). The values of microhardness averaged over each plane of cross-section on the coordinate x along the sample length (**b**).

Figure 5a represents the photograph of the longitudinal section of the sample and the map of microhardness distribution on this section. The right bar demonstrates the color codes of the microhardness values in megapascals. One can see from this map that the highest values of the microhardness occur on the planes symmetrically placed at some distance from the central plane of the sample. This is clearly also seen from the dependence of the values of microhardness averaged over each plane of cross-section on the coordinate *x* along the sample length given in Figure 5b. As one can see from these data, maximum increase in the microhardness occurs not in the central plane where the stress amplitude is maximum, but at a lower value of the stress. The lowest values of microhardness corresponding to the initial state near the specimen edges indicate that the ultrasound effect in these regions was minimal, which is consistent with the zero value of the stress amplitude there. The maximum value of the microhardness measured at points A1 and A2 is equal to 1140 ± 50 MPa.

**Figure 6:**
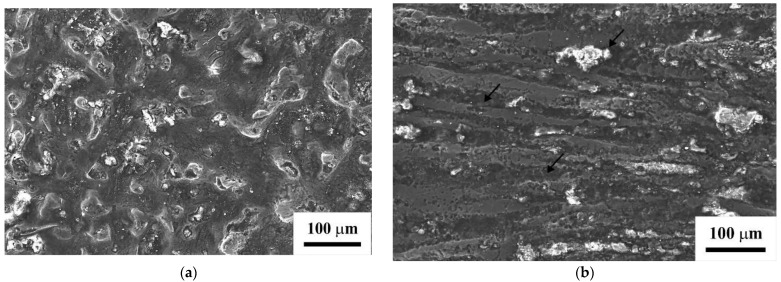

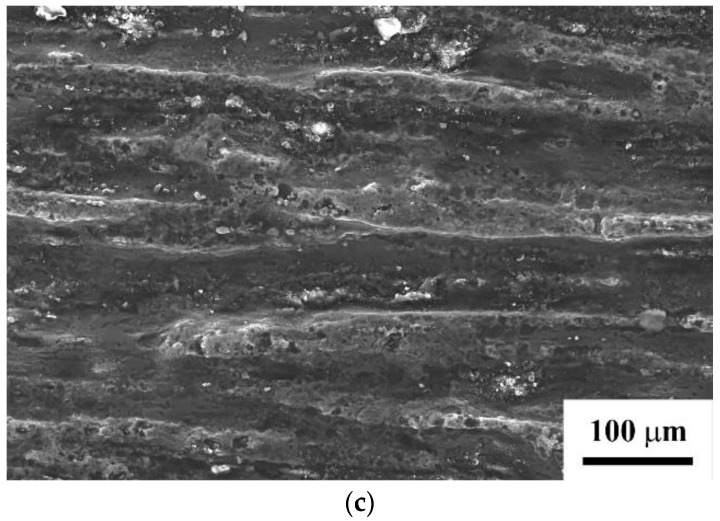
Structure of specimens of Mg-Al-Zn alloy in the initial state (**a**), reduction–ECAP (**b**), reduction–ECAP+UST, (**c**) after corrosion. On the image b, arrows indicate the places of corrosion development.

Studies of the corrosion resistance of the specimens in the initial, severely deformed, and UST-treated states show that the failure of the specimen after reduction with further ECAP occurs most quickly. Figure 6 represents the surface structures of the specimens after two minutes of exposition in a concentrated HCl environment. It can be noted that there are corrosion areas on the specimen surface after reduction–ECAP. Their width is close to the size of the formed grains, and pickling is observed in the initial and UST-treated specimens, predominantly in thin boundaries. The analysis of corrosion studies showed that the depth indicator was 52 mm/year for the initial state, 145 mm/year for the deformed state, and after UST, it was close to the initial state of 58 mm/year.

## 4. Discussion

As for most metals and alloys, refinement of the structure using SPD methods is a perspective trend in obtaining improved properties in magnesium materials. However, a relatively high temperature required for deformation processing reduces the efficiency of using these methods for magnesium alloys. In particular, as the present study shows, ECAP at 350 °C allows for refining the grains, but due to the high activity of recovery processes, prevents achieving a relatively high density of crystal lattice defects. The studies of the effect of post-deformation UST on the magnesium alloy carried out in the present paper show that this method can exert an additional effect on the dislocation structure of the material. On one hand, UST leads to an additional charging of low-angle boundaries by free dislocations that results in the formation of new high-angle boundaries and increases the fraction of this type of boundaries [39]. On the other hand, UST leads to the generation of new dislocations [19,40,41], which is proved by an increased density of dislocations according to the XRD results. Grain boundaries can be a source of new dislocations. The result of such structural changes was an increased tensile strength and yield stress while the nature of the grain structure was preserved, which allowed keeping the relative elongation at 14% corresponding to ECAP treatment.

It should be noted that the effect of ultrasound on the microhardness of the Mg-Al-Zn alloy is similar to the effect on the strength of ultrafine grained nickel processed by ECAP, which was observed in [22]. In the cited work, UST with stress amplitudes in the range from 0 to 100 MPa increased the tensile strength and elongation to failure of ultrafine-grained nickel. The effect was amplitude-dependent and the maximum increase of the strength was achieved not at the center of the sample where the amplitude was equal to 100 MPa, but at the stress amplitude of about 75 MPa (in nickel, this corresponds to the deformation amplitude of 3.7 × 10^−4^). The dependence of the average microhardness in a cross-section on its coordinate found in this paper (Figure 5b) is similar to the dependence of the tensile strength of UFG Ni on the UST amplitude. Therefore, in the case of Mg-Al-Zn alloy, the effect of ultrasound depends on its amplitude and there is an optimal amplitude of the stress which provides the highest increase in the strength.

Another important property of magnesium is corrosion resistance. It is known that magnesium and its alloys dissolve quite actively in hydrochloric acid. A significant effect on corrosion resistance in magnesium alloys is exerted by particles of second phases, the length of grain boundaries, and the characteristics of these boundaries [10,42,43].

Particles of the β phase can play a dual role in corrosion. In a cast alloy, β particles are stable and, when distributed along the network of grain boundaries, they act as a barrier in corrosion propagation [42]. A low concentration of the β phase can act as a galvanic cathode and accelerate corrosion of the magnesium matrix, respectively. According to the findings of the present paper, ECAP results in a decreased fraction of β particles, and ultrasonic treatment almost does not change the fraction of second-phase particles, so it is unlikely that such small changes will lead to a substantial change in the corrosion rate.

Another factor affecting the corrosion rate is related to grain boundaries. It has been noted many times [42,44] that grain refinement and translation of the system into a non-equilibrium state by SPD methods lead to an increased rate of corrosion in general. It is noted [45,46] that low-angle boundaries have higher corrosion resistance than high-angle ones. EBSD analysis shows that the fraction of high-angle boundaries after reduction–ECAP in MA2 alloy is 72%. After ultrasonic treatment, the fraction of high-angle boundaries rises by another 17%, but the corrosion rate goes down, which requires additional explanation. As given in [47,48], it should be noted that the corrosion resistance of a certain grain boundary is affected by its misorientation. After ultrasonic treatment, the share of boundaries with misorientation angles of 27–32° and 86–90° rises (Figure 3c,d). The formation of peaks near 30° and 90° in the misorientation distribution after deformation treatment of magnesium materials has been previously noted [48]. The analysis of the results of the cited paper shows that the peak of about 30° can be primarily related to the boundaries Σ13a corresponding to the axis 〈0001〉, and partially to Σ15a $\langle 2\bar{11}0\rangle$ (Table 4). The rotation axis 〈0001〉 is parallel to axis c, so the boundaries fully overlap [48].

An additional peak near misorientation of 90° is associated with boundaries Σ15b and Σ17a. These boundaries can be assigned to twin boundaries $\left(10\bar{1}2\right)$ [49]. Twins can have a dual effect of the corrosion rate [50]. On the one hand, they can have a negative effect of the corrosion resistance of the material [51]. On the other hand, twins of system $\left(10\bar{1}2\right)$ can increase the corrosion resistance. This is related to the high oxidation activity of atoms in the twin plane as compared with atoms in the densely packed basis plane [52,53], which allows for the easier formation of the oxide film or reduced effects of corrosion anisotropy in misoriented areas in the materials. Twin boundaries are fully coherent and have a lower inter-phase energy than some non-coherent grain boundaries. Twin boundaries and perfect grain boundaries can significantly increase the number of physical barriers for any corrosion action [54,55]. In this manner, an increased fraction of twin boundaries of system $\left(10\bar{1}2\right)$ and of Σ13a boundaries can play an important role in increasing the corrosion resistance of Mg-5.1Al-2.5Zn alloy in hydrochloric acid in the case of equal quantitative characteristics of structure and particles.

## 5. Conclusions

1. Combined deformation processing of Mg-Al-Zn alloy by reduction and ECAP at 350 °C simultaneously lead to a refined structure, with a grain size of 10–20 µm and the occurrence of recrystallized grains 1–2 µm in size. This leads to an increased ultimate tensile strength of 280 ± 10 MPa, while the relative elongation to failure increases up to 14 ± 1%. The enhanced ductility can be related to equiaxed recrystallized grains.
2. Standing-wave UST at 20 kHz and a vibration amplitude of 10 µm has a notable effect on the dislocation structure in the specimen near its center. Along with the increased fraction of large-angle boundaries from 72% to 89%, the high density of dislocations is kept at around 10^14^ m^−2^. There is additional strengthening of the material up to 310 ± 5 MPa while maintaining the relative elongation of 14 ± 1%.
3. The microhardness analysis shows an uneven effect of UST on the specimen structure, which is related to the nature of the standing wave occurrence in the specimen. The maximum increase of 450 MPa in microhardness occurs near the central area and the microhardness reaches 1140 ± 50 MPa.
4. Corrosion studies in hydrochloric acid indicate a substantially decreased corrosion resistance of the Mg-Al-Zn alloy with refined grains and increased dislocation density after reduction–ECAP. Further UST, which leads to an increased fraction of high-angle boundaries, causes an almost two-fold increase in the corrosion resistance of the alloy in HCl at a high density of dislocations. The physical nature of such a behavior can be related to the changes in the characteristics of grain boundaries, namely, an increased fraction of fully overlapping Σ13a boundaries and of twin boundaries of Σ15b and Σ17a systems.

## Figures and Tables

**Table 1:** XRD results.

State	Lattice Parameter, ang.	CSR, nm	Microstrains, %	Density of Dislocations, 10^14^ m^−2^
Initial	3.1955 ± 0.0008 5.1913 ± 0.0005	108	0.15	0.48
Reduction–ECAP	3.2018 ± 0.0005 5.2010 ± 0.0008	56	0.23	3.62
Reduction–ECAP + UST	3.1990 ± 0.0006 5.1994 ± 0.0007	48	0.26	4.24

**Table 2:** Changes in the volumetric share (%) of second phases during reduction–ECAP and UST.

State	Al_12_Mg_17_	MgZn_2_	Mg_4_Zn_7_
Initial	2.81	0.65	0.24
Reduction–ECAP	1.20	0.43	0.19
Reduction–ECAP + UST	1.34	0.51	0.15

**Table 3:** Mechanical properties of Mg-Al-Zn alloy.

State	σ_T_, MPa	σ_0.2_, MPa	Elongation, %
Initial	190 ± 10	120 ± 5	8 ± 1
Reduction–ECAP	280 ± 10	185 ± 10	14 ± 1
Reduction–ECAP + UST	310 ± 5	215 ± 10	14 ± 1

**Table 4:** Possible near-CSL grain boundaries in magnesium [47].

Σ	Axis	Angle	c/a	Remarks
1	Any	0	Any	Low angle boundaries
7	〈0001〉	21.79	Any	Perfect coincidence
9	$\langle 2\bar{11}0\rangle$	56.25	1.620	$\left(10\bar{1}1\right)$ twin
10	$\langle 10\bar{1}0\rangle$	78.46	1.633	
11	$\langle 10\bar{1}0\rangle$	62.96	1.633	$\left(11\bar{2}2\right)$ twin
13a	〈0001〉	27.8	Any	Perfect coincidence
13b	$\langle 10\bar{1}0\rangle$	85.59	1.620	
14	$\langle 10\bar{1}0\rangle$	44.42	1.633	
15a	$\langle 2\bar{11}0\rangle$	29.93	1.620	
15b	$\langle 2\bar{11}0\rangle$	86.18	1.620	$\left(10\bar{1}2\right)$ twin
17a	$\langle 2\bar{11}0\rangle$	86.63	1.633	$\left(10\bar{1}2\right)$ twin
17b	$\langle 10\bar{1}0\rangle$	49.68	1.620	
17c	$\langle 2\bar{11}0\rangle$	49.68	1.604	
18a	$\langle 10\bar{1}0\rangle$	63.62	1.613	$\left(11\bar{2}2\right)$ twin
18b	$\langle 2\bar{11}0\rangle$	70.53	1.633	
19	〈0001〉	13.70	Any	Perfect coincidence
21	$\langle 70\bar{7}2\rangle$	73.40	1.620	
23a	$\langle 2\bar{11}0\rangle$	55.80	1.643	$\left(10\bar{1}1\right)$ twin
23b	$\langle 2\bar{11}0\rangle$	34.30	1.604	
23c	$\langle 2\bar{11}0\rangle$	77.44	1.620	
23d	$\langle 10\bar{1}0\rangle$	34.30	1.620	$\left(11\bar{2}1\right)$ twin
25a	$\langle 2\bar{11}0\rangle$	63.90	1.620	$\left(10\bar{1}3\right)$ twin
25b	$\langle 10\bar{1}0\rangle$	23.07	1.633	

## Data Availability

Not applicable.
